# Assessment of the QuantiFERON-TB Gold In-Tube test for the detection of *Mycobacterium tuberculosis* infection in United States Navy recruits

**DOI:** 10.1371/journal.pone.0177752

**Published:** 2017-05-17

**Authors:** Jason M. Lempp, Margan J. Zajdowicz, Arlene L. Hankinson, Sean R. Toney, Lisa W. Keep, James D. Mancuso, Gerald H. Mazurek

**Affiliations:** 1 Division of Tuberculosis Elimination, Centers for Disease Control and Prevention, Atlanta, Georgia, United States of America; 2 Department of Epidemiology, Rollins School of Public Health, Emory University, Atlanta, Georgia, United States of America; 3 Naval Hospital Great Lakes, Medical Corps, United States Navy, Great Lakes, Illinois, United States of America; 4 Department of Preventive Medicine and Biostatistics, Uniformed Services University of the Health Sciences, Medical Corps, United States Army, Bethesda, Maryland, United States of America; University of Cape Town, SOUTH AFRICA

## Abstract

**Background:**

Immunologic tests such as the tuberculin skin test (TST) and QuantiFERON^®^-TB Gold In-Tube test (QFT-GIT) are designed to detect M*ycobacterium tuberculosis* infection, both latent *M*. *tuberculosis* infection (LTBI) and infection manifesting as active tuberculosis disease (TB). These tests need high specificity to minimize unnecessary treatment and high sensitivity to allow maximum detection and prevention of TB.

**Methods:**

Estimate QFT-GIT specificity, compare QFT-GIT and TST results, and assess factor associations with test discordance among U.S. Navy recruits.

**Results:**

Among 792 subjects with completed TST and QFT-GIT, 42(5.3%) had TST indurations ≥10mm, 23(2.9%) had indurations ≥15mm, 14(1.8%) had positive QFT-GIT results, and 5(0.6%) had indeterminate QFT-GITs. Of 787 subjects with completed TST and determinate QFT-GIT, 510(64.8%) were at low-risk for infection, 277(35.2%) were at increased risk, and none had TB. Among 510 subjects at low-risk (presumed not infected), estimated TST specificity using a 15mm cutoff, 99.0% (95%CI: 98.2–99.9%), and QFT-GIT specificity, 98.8% (95%CI: 97.9–99.8%), were not significantly different (p>0.99). Most discordance was among recruits at increased risk of infection, and most was TST-positive but QFT-GIT-negative discordance. Of 18 recruits with TST ≥15mm but QFT-GIT negative discordance, 14(78%) were at increased risk. TB prevalence in country of birth was the strongest predictor of positive TST results, positive QFT-GIT results, and TST-positive but QFT-GIT-negative discordance. Reactivity to *M*. *avium* purified protein derivative (PPD) was associated with positive TST results and with TST-positive but QFT-GIT-negative discordance using a 10 mm cutoff, but not using a 15 mm cutoff or with QFT-GIT results.

**Conclusions:**

*M*. *tuberculosis* infection prevalence was low, with the vast majority of infection occurring in recruits with recognizable risks. QFT-GIT and TST specificities were high and not significantly different. Negative QFT-GIT results among subjects with TST induration ≥15 mm who were born in countries with high TB prevalence, raise concerns.

## Introduction

Tuberculosis (TB) and *Mycobacterium tuberculosis* transmission increase during periods of military conflict [1,2]. Increases may be due to reactivation of latent *M*. *tuberculosis* infection (LTBI) from stress, malnutrition, or other co-morbidities; disruption of TB treatment and prevention efforts; migration of individuals with contagious disease; and over-crowding [1,3–5]. Military personnel are frequently in conflict settings and may be infected with *M*. *tuberculosis* through interaction with populations with increased TB prevalence [6–9]. Close quarters on Navy ships may facilitate *M*. *tuberculosis* transmission [10,11]. Vigilant screening for both TB and LTBI, and appropriate treatment can limit the spread of infection and reduce operational disruptions [12]. Immunologic tests such as the tuberculin skin test (TST) and interferon gamma (IFN-γ) release assays (IGRAs) can facilitate screening for *M*. *tuberculosis* infection, including both latent infection (i.e., LTBI) and infection manifesting as disease (i.e., TB) [13].

Until 2001, TST was the only commercially-available immunologic test for *M*. *tuberculosis* infection. Documented limitations of TST prompted the development of IGRAs. As *in vitro* blood tests, IGRAs offered logistic advantages including the ability to complete testing after a single patient visit and the ability to rapidly implement methodological improvements. This was not possible with *in vivo* tests like TST. For example, multiple IGRA test antigens could be compared using blood from a single venipuncture while assessment of multiple *in vivo* skin test antigens would require lengthy prerequisite studies documenting the safety of each antigen to be injected.

In 2001, the QuantiFERON^®^-TB test (QFT) (Cellestis Limited, Carnegie, Victoria, Australia) became the first IGRA approved by the Food and Drug Administration (FDA) for the detection of *M*. *tuberculosis* infection [14]. QFT used an enzyme-linked immunosorbent assay (ELISA) to measure the amount of IFN-γ released in response to purified protein derivative (PPD) produced from *M*. *tuberculosis* (tuberculin PPD), compared to the amount released in response to controls [15]. QFT controls included PPD produced from *M*. *avium* (avian PPD) to aid in discriminating *M*. *tuberculosis* infection from nontuberculous mycobacterium (NTM) sensitization. Despite the avian PPD control, QFT specificity was less than TST specificity [16,17].

In an attempt to improve specificity, subsequent generations of IGRAs used manufactured peptides that represent specific *M*. *tuberculosis* antigens such as early secreted antigenic target–6 (ESAT-6) and culture filtrate protein–10 (CFP-10). ESAT-6 and CFP-10 are released by pathogenic *M*. *tuberculosis* and are highly antigenic; they are absent from all Bacillus Calmette–Guérin (BCG) vaccines and most NTM [18–21]. As test antigens, these proteins offer the possibility of more specific detection of *M*. *tuberculosis* infection [22–29]. However, specificity depends on multiple factors in addition to the test antigen, including the cutoffs used to interpret the test and the analytical methods employed to measure IFN-γ concentrations. The QuantiFERON^®^-TB Gold test (QFT-G) (Cellestis Limited, Carnegie, Victoria, Australia) was the first commercial IGRA approved by FDA to measure response to peptide mixtures representing ESAT-6 and CFP-10 [30].

For IGRAs to measure IFN-γ response accurately, fresh blood specimens containing viable white blood cells are needed. This requirement limited use of early IGRAs to facilities in which trained laboratorians could begin testing blood within a few hours of its collection. The QuantiFERON^®^-TB Gold In-Tube test (QFT-GIT) (Cellestis Limited, Carnegie, Victoria, Australia) was developed to address this limitation by allowing incubation of blood in collection tubes that contain antigens or controls [13,31]. QFT-GIT antigens consist of a single mixture of 14 peptides representing ESAT-6, CFP-10 and a third *M*. *tuberculosis* protein, TB7.7. QFT-GIT was approved by the FDA based partly on data described in this manuscript [13,32].

The objectives of this study were to: 1) estimate the prevalence of *M*. *tuberculosis* infection in U.S. Navy recruits based on QFT-GIT results, 2) estimate QFT-GIT specificity among recruits at low risk for *M*. *tuberculosis* infection, 3) identify factors associated with positive QFT-GIT results, and 4) identify factors associated with discordance between QFT-GIT and TST.

## Materials and methods

### Ethics statement and subject selection

This study is part of a larger study of IGRAs, some portions of which have been described previously [27,33–35]. This portion of the study was conducted at the Recruit Training Command (RTC), Great Lakes, Illinois after approval by the Institutional Review Boards of the National Naval Medical Center and the Centers for Disease Control and Prevention (CDC). All U.S. Navy recruits enter boot camp at RTC and have a comprehensive medical assessment with blood collected as part of this exam. All recruits receive a baseline TST, except those with documented prior positive TST results or a history of LTBI or TB treatment. At the time of the study, recruits with TST indurations ≥5 mm and those excluded from TST testing received further evaluation and a chest radiograph [36]. Navy Tuberculosis Control Program policies stipulate risk-based criteria for interpreting TST reactions [37].

Incoming recruits scheduled for TST between January 31 and February 12, 2004, were asked to participate in the parent study [27] and when possible to provide additional blood for QFT-GIT. Written informed consent was obtained and subjects completed a questionnaire about risk for *M*. *tuberculosis* infection, prior TST, BCG vaccination, and symptoms compatible with TB. Chest radiograph, mycobacterial culture, and TB related treatment data were abstracted from medical records. Subjects were categorized as: 1) “tuberculosis suspects” if they reported a cough, fever, or unintentional weight loss of more than 2 weeks duration, or had an abnormal chest radiograph consistent with TB; 2) “increased risk” for *M*. *tuberculosis* infection if they did not meet the “tuberculosis-suspect” criteria, but reported contact with someone with TB, birth (or residence >1 month) in a country where estimated TB prevalence exceeded 20 cases per 100,000 population [38], or having resided, worked, or volunteered >1 month in a homeless shelter, prison, drug rehabilitation unit, hospital, or nursing home; or 3) “low risk” for *M*. *tuberculosis* infection if they were neither suspects nor at increased risk. Data from subjects with previously treated TB or LTBI, subjects classified as TB suspects, and subjects whose risk of infection could not be classified were excluded from analysis.

### Test methods

Blood for QFT-GIT was collected after blood was collected for other routine and investigational tests (including QFT and QFT-G) and prior to applying a TST. TST, QFT, and QFT-G methods and results from a portion of the subjects included in this study have been reported previously [27]. For QFT-GIT, approximately 1 mL of blood was collected in tubes containing heparin alone (Nil tube); heparin, dextrose, and phytohemagglutinin (PHA) (Mitogen tube); and heparin, dextrose, and a single mixture of peptides representing ESAT-6, CFP-10, and part of TB7.7 (TB Antigen tube). Blood was mixed with the tube contents and, within 12 hours of collection, incubated (16 to 24 hours at 37°C), centrifuged, and the plasma harvested. The concentration of IFN-γ in 50 μl of each plasma sample was determined by ELISA as previously described for QFT-G [33,34]. The Mitogen Response was calculated by subtracting the IFN-γ concentration in plasma from unstimulated blood (Nil) from the IFN-γ concentration in plasma from mitogen stimulated blood. The TB Response was calculated by subtracting Nil from the IFN-γ concentration in plasma from blood stimulated with the mixture of peptides representing ESAT-6, CFP10, and TB7.7. QFT-GIT was performed within the limitations stipulated in original and subsequent package inserts [32,39]. QFT-GIT was interpreted as described in published guidelines [13]. TST interpretation was stratified by risk according to published guidelines [40], unless otherwise stated that the cutoff for a positive reaction was 15 mm or 10 mm.

### Statistical methods

Statistical analyses were conducted using SAS (Ver. 9.2, SAS Institute, Cary, NC, USA) and IBM SPSS Statistics for Windows (Version 21.0, IBM Corp, Armonk, NY, USA). Categorical variables were compared using Fisher’s exact test and distributional differences in continuous measures between groups of subjects were assessed using the Wilcoxon rank sum exact test. P-values ≤0.05 were considered significant. Prevalence estimates were based on subjects who completed both TST and QFT-GIT. Subjects categorized as “low risk” were assumed to be uninfected, and specificities were calculated among low-risk subjects with completed TST and determinate QFT-GIT results. Estimates of specificity (and prevalence) were compared using McNemar’s exact test. Overall test agreement was calculated as the number of subjects with concordant results divided by the total number tested, excluding subjects with incomplete TST or indeterminate QFT-GIT results; positive agreement was calculated as the number of subjects with positive results for both tests divided by the number of subjects with positive results to either test; agreement beyond chance was assessed with Cohen’s Kappa statistic (*k*) [41].

Discordance was categorized as “TST-positive but QFT-GIT-negative” or “TST-negative but QFT-GIT-positive” using a 10 or 15 mm cutoff for TST. Subjects in each category of discordance were compared to those with concordant results. Bivariate analyses were used to identify factors associated with positive TST or QFT-GIT results, and with each type of discordance using logistic regression. Factors evaluated included age, sex, race/ethnicity, TB prevalence in country of birth, TB prevalence in countries of residence ≥1 month other than place of birth, history of exposure to someone with TB, ≥1 month residence or employment in a congregate living facility with increased risk of *M*. *tuberculosis* exposure (hospital, nursing home, homeless shelter, drug rehabilitation unit, prison, or jail), self-reported BCG vaccination status, TST placed in the prior year, and reactivity to *M*. *avium* PPD. Prevalence of TB by country of birth and residence were categorized as low “<20 cases per 100,000 population”, medium “20 through 100 cases per 100,000 population”, or high “>100 cases per 100,000 population” based on World Health Organization (WHO) estimates for 1990 [38]. Subjects were classified as having avian PPD reactivity if QFT was interpreted as “negative for *M*. *tuberculosis* infection with avian PPD reactivity”; all other subjects were classified as having no evidence of avian PPD reactivity.

Multivariate logistic regression models were employed to identify factors associated with test results and test discordance using backwards elimination. Collinearity between variables included in the models was assessed using Pearson’s correlation coefficients (R) and variance inflation factor (VIF) values.

## Results

**Fig 1:**
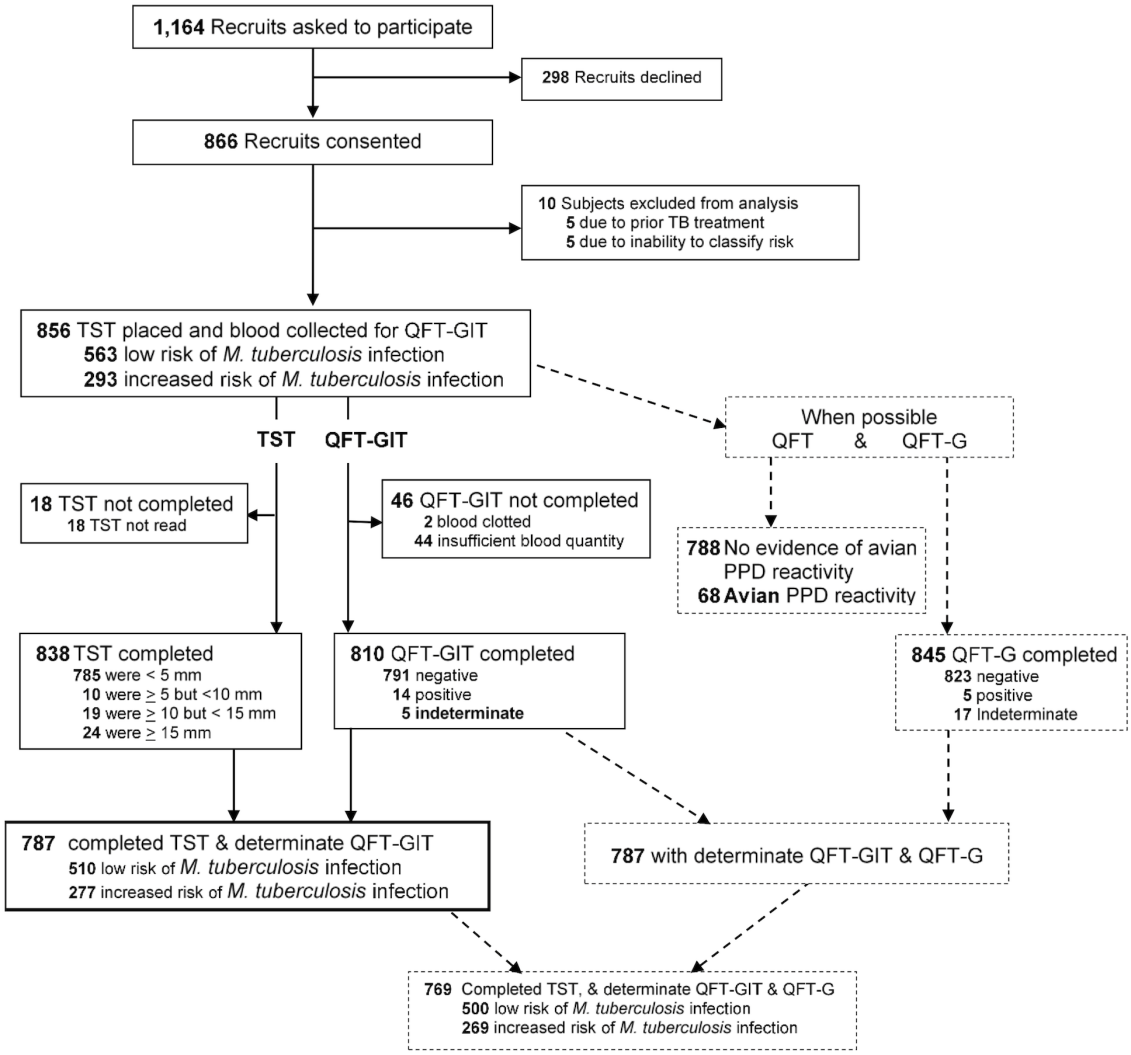
Diagram of study participants and testing. QFT = QuantiFERON^®^-TB test; QFT-G = QuantiFERON^®^-TB Gold test; QFT-GIT = QuantiFERON^®^-TB Gold In-Tube test; TST = tuberculin skin test.

Of 1,164 recruits asked to participate, 866 (73%) consented and of these 10 were excluded for the reasons indicated in Fig 1. The outcomes of TST and QFT-GIT testing for the 856 eligible subjects who had TST placed and blood collected are depicted in Fig 1 and Table 1. TST induration was ≥5 mm for 53 subjects of whom all received a chest radiograph and all radiographs were interpreted as normal. None of the participants were suspected to have TB. Both TST and QFT-GIT were completed for 792 subjects. Test results using various criteria for this and other cohort subsets are shown in Table 2. Estimated prevalence using QFT-GIT (1.8%) was lower than by TST using risk-stratified interpretation (4.8%; p<0.01) or a 10 mm cutoff (5.3%; p<0.01), but not significantly different than by TST using a 15 mm cutoff (2.9%; p = 0.12).

**Table 1:** Outcomes of the QuantiFERON^®^-TB Gold In-Tube test versus the tuberculin skin test among 856 U.S. Navy recruits who had blood collected and skin test placed.

		QFT-GIT results				
TST Results	Recruit Category ^a^	Negative	Positive	Indeterminate	Incomplete	All
< 5 mm	All	726	9	5	45	785
	Low Risk	489	5	3	38	535
	Increased Risk	237	4	2	7	250
≥ 5 and < 10 mm	All	10	0	0	0	10
	Low Risk	7	0	0	0	7
	Increased Risk	3	0	0	0	3
≥ 10 and < 15 mm	All	19	0	0	0	19
	Low Risk	4	0	0	0	4
	Increased Risk	15	0	0	0	15
≥ 15 mm	All	18	5	0	1	24
	Low Risk	4	1	0	0	5
	Increased Risk	14	4	0	1	19
Incomplete	All	18	0	0	0	18
	Low Risk	12	0	0	0	12
	Increased Risk	6	0	0	0	6
All	All	791	14	5	46	856
	Low Risk	516	6	3	38	563
	Increased Risk	275	8	2	8	293

QFT-GIT = QuantiFERON^®^-TB Gold In-Tube test; TST = tuberculin skin test.

^a^ Navy recruits were categorized as having an “increased risk” for *M*. *tuberculosis* infection if they did not meet the “tuberculosis-suspect” criteria, but reported contact with someone with TB, birth (or residence >1 month) in a country where estimated TB prevalence exceeded 20 cases per 100,000 population, or having resided, worked, or volunteered >1 month in a homeless shelter, jail, prison, drug rehabilitation unit, hospital, or nursing home; or as having a “low risk” for *M*. *tuberculosis* infection if they were neither suspects nor at increased risk.

**Table 2:** Results for various cohort subsets.

Cohort Subset Size & Description	Characteristic	Value
792 subjects with completed TST and QFT-GIT	QFT-GIT Positive	14 (1.8%)
	TST > = 15	23 (2.9%)
	TST > = 10	42 (5.3%)
	TST Positive by Risk Stratified Interpretation	38 (4.8%)
	QFT-GIT Indeterminate	5 (0.6%)
787 subjects with completed TST and determinate QFT-GIT	Low risk of *M*. *tuberculosis* infection	510 (64.8%)
	Increased risk of *M*. *tuberculosis* infection	277 (35.2%)
277 subjects at increased risk with completed TST and determinate QFT-GIT	QFT-GIT Positive	8 (2.9%)
	TST > = 15	18 (6.5%)
	TST > = 10	33 (11.9%)
510 subjects at low risk with completed TST and determinate QFT-GIT	QFT-GIT Positive	6 (1.2%)
	TST > = 15	5 (1.0%)
	TST > = 10	9 (1.8%)
	QFT-GIT Negative	504 (98.8%)
	TST < 15	505 (99.0%)
	TST < 10	501 (98.2%)
807 subjects who had QFT-GIT and QFT-G completed	QFT-GIT positive	14 (1.7%)
	QFT-G positive	5 (0.6%)
	QFT-GIT indeterminate	5 (0.6%)
	QFT-G indeterminate	16 (2.0%)
787 subjects with determinate QFT-GIT and QFT-G	QFT-GIT positive	11 (1.4%)
	QFT-G positive	5 (0.6%)
769 subjects with competed TST and determinate QFT-GIT and QFT-G results	Low risk	500 (65.0%)
500 subjects at low risk with determinate QFT-G, QFT-GIT, and TST results	QFT-GIT negative	497 (99.4%)
	QFT-G negative	499 (99.8%)
	TST <15	495 (99.0%)
	TST <10	491 (98.2%)

TST = tuberculin skin test; QFT-GIT = QuantiFERON^®^-TB Gold In-Tube test; QFT-G = QuantiFERON^®^-TB Gold test.

As shown in Table 2, among the 787 subjects with completed TST and determinate QFT-GIT results, 510 (64.8%) were categorized as “low risk” and 277 (35.2%) were categorized as “increased risk” for *M*. *tuberculosis* infection. TB Response by QFT-GIT ranged from -1.51 to 12.29 IU/mL. Positive QFT-GIT results were not significantly more frequent among subjects at increased risk than among subjects at low risk (2.9% versus 1.2%; p = 0.10) and TB Response was not significantly greater (Z = -0.43; p = 0.67). TST induration was observed in 52 subjects who had determinate QFT-GIT results, and ranged from 6 to 50 mm. Positive TST results using a 10 mm cutoff were more frequent among subjects at increased risk than among subjects at low risk (11.9% vs 1.8%; p<0.01). Similarly, TST results ≥15 mm were more frequent among subjects at increased risk than among those at low risk (6.5% vs 1.0%; p<0.01). Induration size was significantly larger in recruits at increased risk than in recruits at low risk (Z = -5.42; p<0.01). Measures of agreement between QFT-GIT and TST (using various interpretation criteria) are shown in Table 3.

**Table 3:** Test agreement.

	Overall Agreement	Kappa	Positive Agreement	Negative Agreement
QFT-GIT vs. TST with 10 mm cutoff ^a^	741/787 (94.2%)	0.16	5/51 (9.8%)	736/782 (94.1%)
QFT-GIT vs. TST with 15 mm cutoff ^a^	760/787 (96.6%)	0.25	5/32 (15.6%)	755/782 (96.5%)
QFT-GIT vs. TST with risk stratified interpretation ^a^	745/787 (94.7%)	0.17	5/47 (10.6%	740/782 (94.6%)
QFT-GIT vs. QFT-G ^b^	781/787 (99.2%)	0.62	5/11 (45.5%)	776/782 (99.2%)

^a^ Among 787 subjects with completed TST and determinate QFT-GIT.

^b^ Among 787 subjects with determinate QFT-GIT and QFT-G results.

Among the 510 subjects at low risk with completed TST and determinate QFT-GIT, calculated QFT-GIT specificity was 98.8% (95% CI = 97.9–99.8%). Calculated TST specificity was 99.0% (95% CI = 98.2–99.9%) using a 15 mm cutoff, but 98.2% (95% CI = 97.1–99.4%) using a 10 mm cutoff. The differences between QFT-GIT specificity and TST specificity (using either a 10 or 15 mm cutoff) were not significant (p = 0.58 and >0.99, respectively).

QFT-GIT and QFT-G were completed for 807 subjects and frequencies of test results for this cohort subset are shown in Table 2. The outcomes of QFT-GIT and QFT-G are compared in S1 Table. The prevalence estimate by QFT-G (0.6%) was lower than that by QFT-GIT (1.7%; p<0.01). Among the 807 subjects who had QFT-GIT and QFT-G completed, QFT-GIT gave less frequent indeterminate results (0.6% versus 2.0%; p = 0.02). Measures of agreement between QFT-GIT and QFT-G are shown in Table 3.

**Fig 2:**
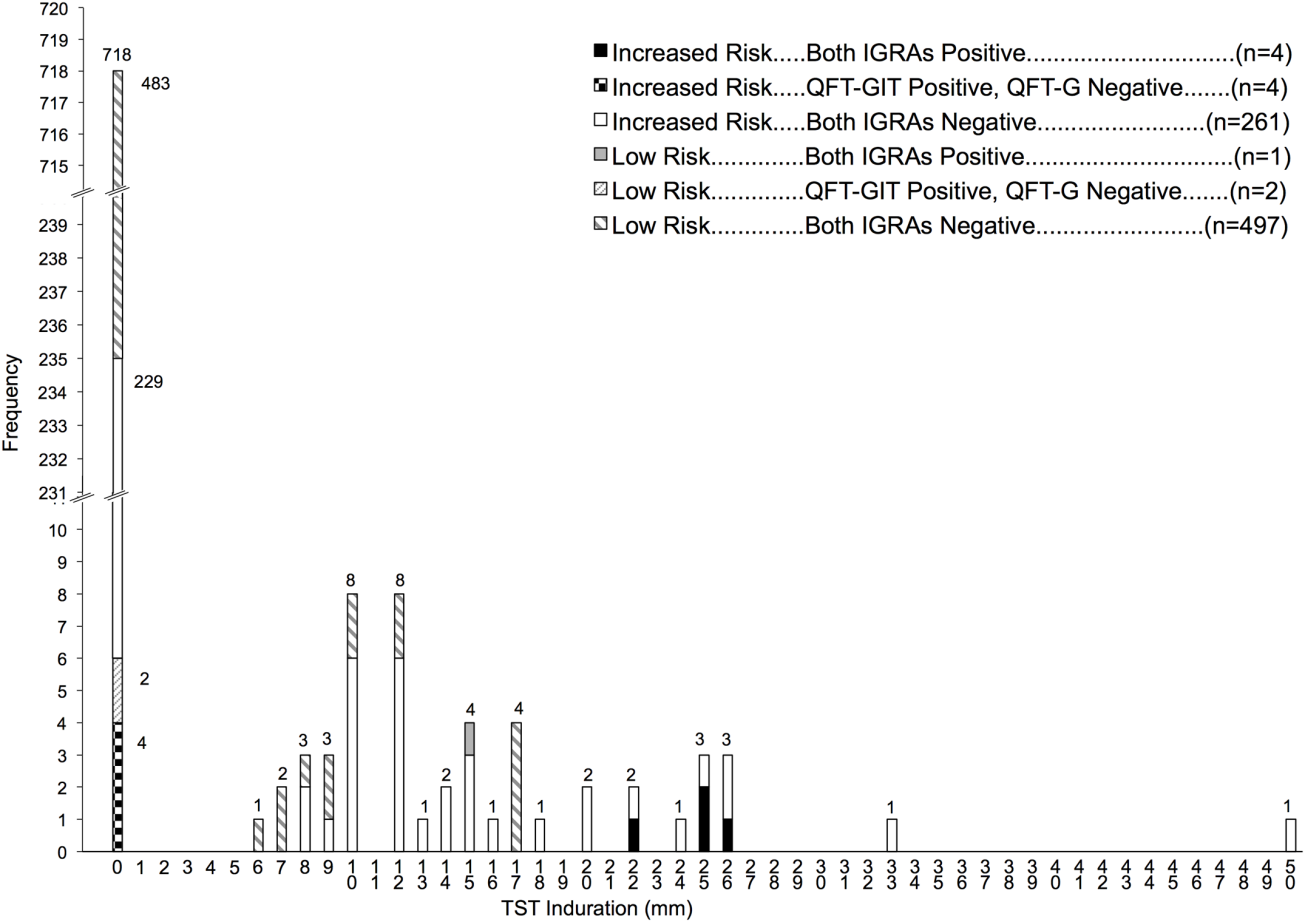
Comparison of QuantiFERON^®^-TB Gold In-Tube test, tuberculin skin test, and QuantiFERON^®^-TB Gold test results among 769 U.S. Navy recruits categorized by risk of *M*. *tuberculosis* infection. The 769 Navy recruits who had TST, QFT-G, and QFT-GIT completed with determinate test results were categorized as having an “increased risk” for *M*. *tuberculosis* infection if they did not meet the “tuberculosis-suspect” criteria, but reported contact with someone with TB, birth (or residence >1 month) in a country where estimated TB prevalence in 1990 exceeded 20 cases per 100,000 population, or having resided, worked, or volunteered >1 month in a homeless shelter, prison, drug rehabilitation unit, hospital, or nursing home; or as having a “low risk” for *M*. *tuberculosis* infection if they were neither suspects nor at increased risk. IGRAs = interferon gamma release assays; QFT-GIT = QuantiFERON^®^-TB Gold In-Tube test; QFT-G = QuantiFERON^®^-TB Gold test; TST = tuberculin skin test.

Among the 769 subjects with completed TST and determinate QFT-GIT and QFT-G results, 5 were positive by all three tests, with TST indurations ranging from 15 to 26 mm (Fig 2) and TB Responses ranging from 0.39 to 12.29 for QFT-GIT and from 0.46 to 4.87 for QFT-G. Of these 5 subjects, 4 were at increased risk of *M*. *tuberculosis* infection and one was at low risk. The low-risk recruit’s TST induration was 15 mm while his TB Response was 0.39 IU/mL by QFT-GIT and 0.46 by QFT-G. Of the 42 subjects with TST induration ≥10 mm, 37 (88.1%) were negative by both IGRAs, and 29 (69.0%) of these were at increased risk.

Among 500 low-risk subjects with completed TST and determinate QFT-G and QFT-GIT, estimated QFT-G specificity was 99.8% (95% CI = 99.4–99.9%) and QFT-GIT specificity was 99.4% (95% CI = 98.7–99.9%) (p = 0.50). While estimated QFT-GIT specificity increased from 98.8 to 99.4%, TST specificity estimates were unchanged by exclusion of the 10 recruits with incomplete or indeterminate QFT-G results.

Characteristics associated in multivariate analysis with TST induration ≥15 mm, ≥10 mm, and positive QFT-GIT results, are shown in Table 4 (while results of bivariate analysis are shown in S2 Table). For example, the adjusted odds of a positive QFT-GIT were 7.0 times greater for subjects born in high-TB prevalence countries compared to those born in low-TB prevalence countries after controlling for age, race/ethnicity, TB prevalence in country of residence (other than birth), and BCG vaccination status. Collinearity did not appear to affect our assessment in that none of the variables included in our models were highly correlated (all R values ≤0.5) and all VIF values were <2.

**Table 4:** Associations between selected subject characteristics and tuberculin skin test or QuantiFERON^®^-TB Gold In-Tube test results.

		TST ≥ 15 mm		Positive QFT-GIT		TST ≥ 10 mm^a^	
Characteristic	N	n	aOR (95% CI)	n	aOR (95% CI)	n	aOR (95% CI)
Age ^b^	787	23	**1.2 (1.0–1.3)**	14	Not retained	42	**1.1 (1.0–1.2)**
TB prevalence in country of birth							
<20 cases per 100,000 pop.	713	6	1.0	9	1.0	16	1.0
20–100 cases per 100,000 pop.	23	3	**14.4 (3.2–64.2)**	1	3.6 (0.4–29.3)	5	**12.4 (3.9–39.6)**
>100 cases per 100,000 pop.	51	14	**38.6 (13.7–109.0)**	4	**7.0 (2.1–23.5)**	21	**34.7 (15.2–79.2)**
Reactivity to *M*. *avium* PPD							
No	725	19	Not retained	12	Not retained	32	1.0
Yes	62	4		2		10	**6.8 (2.6–17.5)**

N = total number of recruits with completed test and determinate results; n = the number or recruits with positive test results; TST = tuberculin skin test; QFT-GIT = QuantiFERON^®^-TB Gold In-Tube test; aOR (95% CI) = adjusted Odds Ratios (95% confidence intervals) with boldface font indicating statistically significant differences; TB = tuberculosis.

^a^ TST induration >10 mm includes reactions >15 mm;

^b^ Increase in odds for each year of age.

Of the 42 subjects with discordance between QFT-GIT and TST using a risk-stratified interpretation, 33 (79%) were at increased risk, and 33 (79%) had TST-positive but QFT-GIT negative discordance. While 18 subjects had TST induration ≥15 mm but a negative QFT-GIT result, 37 subjects had TST induration ≥10 mm but a negative QFT-GIT result. Characteristics associated in multivariate analysis with discordance between QFT-GIT results and TST interpretations using a 10 mm or 15 mm cutoff are shown in Table 5 (while results of bivariate analysis are shown in S3 Table). The multivariate model retained age and TB prevalence in country of birth. *M*. *avian* PPD reactivity was associated with discordance using the 10 mm but not the 15 mm cutoff.

**Table 5:** Associations between selected subject characteristics and discordant QuantiFERON^®^-TB Gold In-Tube test and tuberculin skin test results using either a 15 mm or 10 mm cutoff.

	TST ≥ 15 mm but negative QFT-GIT			TST ≥ 10 mm but negative QFT-GIT			TST <15 mm but positive QFT-GIT ^a^		
Characteristic	n ^Concord^	n ^Discord^	aOR (95% CI)	n ^Concord^	n ^Discord^	aOR (95% CI)	n ^Concord^	n ^Discord^	OR (95% CI)
Age ^b^	760	18	**1.2 (1.0–1.3)**	741	37	1.1 (1.0–1.2)	760	9	Not retained
TB prevalence in birth country									
<20 cases per 100,000	700	5	1.0	690	15	1.0	700	8	Not retained
20–100 cases per 100,000	19	3	**18.0 (3.9–83.8)**	17	5	**13.5 (4.2–43.6)**	19	1	
>100 cases per 100,000	41	10	**27.8 (8.8–88.0)**	34	17	**24.2 (10.4–56.4)**	41	0	
Reactivity to *M*. *avium* PPD									
No	702	15	Not retained	689	28	1.0	702	8	Not retained
Yes	58	3		52	9	**6.2 (2.4–16.1)**	58	1	

TST = tuberculin skin test; QFT-GIT = QuantiFERON^®^-TB Gold In-Tube test; n ^Concord^ = the number of subjects with concordant TST (at indicated cutoff) and QFT-GIT results; n ^Discord^ = the number of subjects with discordant TST (at indicated cutoff) and QFT-GIT results; aOR (95% CI) = adjusted Odds Ratios (95% confidence intervals) with boldface font indicating statistically significant differences; TB = tuberculosis.

^a^ TST induration was 0 mm for all subjects with TST <15 mm but positive QFT-GIT results so that all had TST-negative but QFT-GIT-positive discordance regardless of the TST cutoff used;

^b^ Increase in odds for each year of age.

Nine subjects had TST induration <15 but positive QFT-GIT results (Table 1). In each case TST induration was 0 mm, so the TST cutoff used did not affect agreement. None of the subject characteristics examined were associated with this discordance.

## Discussion

This study of U.S. Navy recruits compares the outcome of QFT-GIT to other tests for *M*. *tuberculosis* infection. It supplements previously published comparisons of QFT and QFT-G with TST in almost the same cohort [27], and provides a unique opportunity to assess the effect of *M*. *avium* PPD reactivity on QFT-GIT as measured by older IGRAs that are no longer commercially available. This study confirms that the prevalence of *M*. *tuberculosis* infection among U.S. Navy recruits is low regardless of the test used. Results were positive for 1.8% of recruits by QFT-GIT, 0.6% by QFT-G, and 4.8% by TST using a risk-stratified interpretation. The observed differences highlight the need to find and validate tests that accurately detect *M*. *tuberculosis* infection, differentiate ongoing from resolved infection, and distinguish latent infection from infection manifesting as active disease. Our estimate of prevalence among U.S. Navy recruits based on risk-stratified TST interpretation is similar to the 4.7% prevalence reported for the general non-institutionalized U.S. population using a 10 mm TST cutoff [42]. Adjustments for age, foreign birth, and TST interpretation suggest that infection prevalence may be slightly higher among Navy recruits than the matched U.S. population. However, estimates based on QFT-GIT indicate a lower prevalence of infection among Navy recruits (1.8%) compared to the general U.S. population (5.0%).

We observed relatively high overall agreement between QFT-GIT and TST (94 to 97% with variation due to difference in TST interpretation criteria), but poor positive agreement (10% to 16%) and poor agreement beyond chance (*k* ranged from 0.16 to 0.25). The majority of discordance was among recruits at increased risk of infection. The apparent paradox of high overall agreement and low *k* may be explained partially by the infrequency of positive QFT-GIT results among the study population [41,43]. This does not explain the low positive agreement or explain why the majority of discordance was among recruits at increased risk. Disagreement in test results is ultimately attributable to differences in antigens and test methods. QFT-GIT and QFT-G had higher agreement (*k* = 0.62) than either had compared to TST. Despite methodological similarities in the IGRAs, of the 14 subjects positive by either IGRA, only 5 (36%) were positive by both tests (S1 Table).

QFT-GIT had fewer indeterminate results than QFT-G (0.6% versus 2.0%). The proportion of indeterminate QFT-GIT results was less than expected based on the results of some studies [44,45]. The criteria for results to be indeterminate are not the same for QFT-GIT as QFT-G [13]. For QFT-GIT, Nil values >0.7 IU/mL but ≤8.0 IU/mL can produce a negative result while such values for QFT-G would likely be interpreted as indeterminate. In addition, TB Response values ≥0.35 IU/mL, and ≥25% but <50% of the Nil are interpreted as positive by QFT-GIT if Nil is <8.0 IU/mL, but such results are indeterminate by QFT-G. Allowance of higher [Nil] values decreases the number of indeterminate QFT-GIT results. Characteristics associated in other studies with indeterminate results such as advanced age, underlying disease, or depressed immune status [45–49], are unlikely among young military recruits. Pre-analytic factors may affect indeterminate rates. For this study, blood for QFT-GIT and QFT-G were collected at the same time, and plasma from the same person was analyzed on the same ELISA plate for both QFT-GIT and QFT-G. Therefore, differences in indeterminate rates, and test outcome in general, are likely due to differences in blood stimulation and test interpretation.

We observed no significant difference in estimates of specificity for QFT-GIT (98.8%) and TST (99.0%). Our estimate of QFT-GIT specificity is similar to that found by others conducting studies in low-risk populations [50–52]. Excluding 10 low-risk subjects with indeterminate or incomplete QFT-G results led to specificity estimates of 99.4% for QFT-GIT and 99.8% for QFT-G, which were not significantly different. Interestingly, 3 of the 9 low-risk subjects with indeterminate QFT-G results were positive by QFT-GIT as compared to 3 of 500 with determinate QFT-G results (p<0.01). This suggests that the less stringent criteria for defining indeterminate QFT-GIT results may lower QFT-GIT specificity.

TB prevalence in the country of birth was the strongest predictor of TST results, QFT-GIT results, and discordant TST-positive but QFT-GIT-negative results. Subjects born in high TB prevalence countries were 7 times more likely to have a positive QFT-GIT result, 39 times more likely to have a TST induration ≥15 mm, and 28 times more likely to have TST positive (≥15 mm) but QFT-GIT negative discordant results than subjects born in low-TB prevalence countries. This is particularly worrisome because other studies have shown that birth in countries with high-TB prevalence is strongly associated with risk of developing TB [53,54]. We observed a dose response such that the odds ratios for those born in intermediate prevalence countries were between those born in high and low prevalence countries. Negative QFT-GIT results among subjects with TST induration ≥15 mm who were born in countries with high TB prevalence raises concerns for false-negative QFT-GIT results. While these observations do not exclude the possibility that some recruits with negative QFT-GIT results have false-positive TSTs, the high specificity seen with both tests (especially with TST using a 15mm cutoff), and the preponderance of discordance in recruits at increased risk, justifies concern for false-negative test results.

Understanding disagreement between tests for *M*. *tuberculosis* infection may help clinicians avoid diagnostic errors. Some investigators have attributed TST-positive but QFT-GIT-negative discordance to false-positive TST results following BCG vaccination and NTM exposure [52,55,56]. We observed some associations between TST results and a history of BCG vaccination and reactivity to avian PPD; but these factors were not associated with positive QFT-GIT results. While BCG vaccination status was associated with TST results, and with TST-positive but QFT-GIT-negative discordance using either a 10 mm or 15 mm cutoff in bivariate analysis, BCG vaccination was not significantly associated using either cutoff after controlling for other risks. Forcing BCG status into the models did not meaningfully change the magnitude of associations observed (data not shown). BCG may have a larger effect on TST in populations with a greater number of people vaccinated, especially if vaccinated repeatedly or after 1 year of age [57]. Recall bias can decrease the accuracy of assessments of BCG vaccination status and may have affected our assessments of associations. While BCG is used predominantly in populations at increased risk for *M*. *tuberculosis* infection, BCG vaccination coverage is not directly correlated with TB prevalence. In 1990, when most recruits in this study were born, the average BCG coverage among countries with TB prevalence >100 per 100,000 population was 81%, and less than the 90% average for countries with TB prevalence of 20 to 100 per 100,000 population. Thus, attributing TST-positive but QFT-GIT-negative discordance (that increases consistently with TB prevalence but not BCG coverage) to BCG vaccination may not be appropriate, especially for those with large TST reactions from high prevalence countries.

Reactivity to *M*. *avium* PPD was associated with positive TST results, and with TST-positive but QFT-GIT-negative discordance in both univariate and multivariate analyses using a 10 mm cutoff, but not using a 15 mm cutoff. Several studies among low-risk U.S. Navy recruits, U.S. Army recruits, and healthcare workers demonstrated similar findings, suggesting that NTM sensitization may cause false-positive TST results, especially when using cutoffs <15 mm [27,52,58,59]. In other studies, IGRAs using ESAT-6 and CFP-10 as antigens have been negative despite culture-confirmed infections with NTM [55,60]. Our findings support the hypothesis that NTM sensitization contributes to false-positive TST results and to discordance between QFT-GIT and TST using a 10 mm cutoff but not a 15 mm cutoff.

TST negative but QFT-GIT positive discordance occurred less frequently than TST-positive but QFT-GIT-negative discordance (9 versus 33 using risk-stratified TST interpretation), and no subject characteristics examined were associated with this discordance. While studies in similar healthy populations failed to identify associations with this type of discordance [27,28,52], studies including subjects with immunosuppression, young or advanced age, and severe or chronic illness demonstrate associations between these conditions and TST-negative but QFT-GIT-positive discordance [13,49,61,62].

Using risk-stratified interpretation of TST, 38 recruits in this study would have been diagnosed with LTBI and likely prescribed preventive treatment. With QFT-GIT, 14 recruits would have been candidates for preventive treatment, a reduction of 63%. However, 9 of the 14 subjects with a positive QFT-GIT had a negative TST with induration of 0 mm. Conversely, QFT-GIT would not have detected 10 of 15 subjects considered to be at greatest risk of infectionI, e.g. those who had a TST induration ≥15 mm and were born in high-TB prevalence countries. Reaction sizes of this magnitude are unlikely to result from BCG given once in infancy, or from NTM exposure. [57]

Lack of a diagnostic reference standard to confirm the most common form of *M*. *tuberculosis* infection (i.e., LTBI) and the inability of immunologic tests to differentiate active disease from latent infection, limits assessments of accuracy of tests for *M*. *tuberculosis* infection. One approach to address these diagnostic limitations is to estimate specificity in persons at low risk of infection who are presumed uninfected by *M*. *tuberculosis* [13]. Another approach is to examine factors associated with test positivity and discordance in test results [27,63]. We assumed that subjects with no reported risk were uninfected. However, one low-risk subject was found to have positive QFT-GIT, QFT-G, and TST results (with 15 mm of induration), suggesting that he actually was infected. Although not stipulated in our analytic plan a priori, exclusion of this subject would have increased our specificity estimates.

This study was limited by a relatively small sample size such that exclusion of any subjects with positive results by QFT-GIT, QFT-G, or TST could affect our assessment of associations and specificity. Due to the small sample of subjects with outcomes of interest, multivariate models need to be interpreted with caution. Interaction terms could not be reliably assessed due to the low frequency of positive QFT-GIT results. Requiring complete and determinate results by all three tests was shown to affect the estimate of QFT-GIT specificity which increased from 98.8% to 99.4% when 10 subjects with missing or indeterminate QFT-G were excluded. Although this study was relatively small, the low-risk subjects contribute significantly to prior published assessments of QFT-GIT specificity [50–52]. While enrollment was limited to Navy recruits, the recruits originated from across the U.S. and from other countries making conclusions more generalizable to other U.S. populations of young adults.

## Conclusion

Overall, U.S. Navy recruits have a low measured prevalence of *M*. *tuberculosis* infection regardless of the assay used to detect infection, with the vast majority of infection occurring in recruits with recognizable risks. The specificity of QFT-GIT was high, approaching 99%, with no significant difference from TST or QFT-G specificity. TST results, QFT-GIT results, and TST-positive but QFT-GIT-negative discordance were most strongly associated with TB prevalence in the country of birth. Negative QFT-GIT results among subjects with TST induration ≥15 mm who were born in countries with high TB prevalence raises concerns.

## Supporting information

S1 Table QuantiFERON^®^-TB Gold In-Tube test versus QuantiFERON^®^-TB Gold test. Outcome of testing among 856 Navy recruits who had blood collected and skin test placed.
(DOCX)

S2 Table Associations between selected subject characteristics and tuberculin skin test or QuantiFERON^®^-TB Gold In-Tube test results. (DOCX)

S3 Table Associations between selected subject characteristics and discordant QuantiFERON^®^-TB Gold In-Tube test and tuberculin skin test results using either a 15 mm or 10 mm cutoff. (DOCX)
