# Supplementary material for: Assessment of the QuantiFERON-TB Gold In-Tube test for the detection of Mycobacterium tuberculosis infection in United States Navy recruits
Source: PLoS One. 2017 May 17;12(5):e0177752. doi: 10.1371/journal.pone.0177752 (PMC5435309; doi:10.1371/journal.pone.0177752)
Supplement: S1 Table — Outcome of testing among 856 Navy recruits who had blood collected and skin test placed. (DOCX) [file pone.0177752.s001.docx]

**S1 Table. QuantiFERON^®^-TB Gold In-Tube test versus QuantiFERON^®^-TB Gold test.** Outcome of testing among 856 Navy recruits who had blood collected and skin test placed.

|  | **QFT-GIT results** | | | | |
| --- | --- | --- | --- | --- | --- |
| **QFT-G Results** | Negative | Positive | Indeterminate | Incomplete | Total |
| Negative | 776 | 6 | 4 | 37 | 823 |
| Positive | 0 | 5 | 0 | 0 | 5 |
| Indeterminate | 12 | 3 | 1 | 1 | 17 |
| Incomplete | 3 | 0 | 0 | 8 | 11 |
| Total | 791 | 14 | 5 | 46 | 856 |

QFT-GIT = QuantiFERON^®^-TB Gold In-Tube test; QFT-G = QuantiFERON^®^-TB Gold test.
