# Supplementary material for: Assessment of the QuantiFERON-TB Gold In-Tube test for the detection of Mycobacterium tuberculosis infection in United States Navy recruits
Source: PLoS One. 2017 May 17;12(5):e0177752. doi: 10.1371/journal.pone.0177752 (PMC5435309; doi:10.1371/journal.pone.0177752)
Supplement: S2 Table — (DOCX) [file pone.0177752.s002.docx]

**S2 Table. Associations between selected subject characteristics and tuberculin skin test or QuantiFERON^®^-TB Gold In-Tube test results.**

|  |  | TST > 15 mm | | | | | Positive QFT-GIT | | | | | TST > 10 mm^a^ | | | | |  |
| --- | --- | --- | --- | --- | --- | --- | --- | --- | --- | --- | --- | --- | --- | --- | --- | --- | --- |
| Characteristic | N | n | OR (95% CI) | | aOR (95% CI) | | n | OR (95% CI) | | aOR (95% CI) | | n | OR (95% CI) | | aOR (95% CI) | |  |
| Age ^b^ | 787 | 23 | **1.2 (1.1-1.3)** | | **1.2 (1.0-1.3)** | | 14 | 1.1 (1.0-1.3) | | Not retained | | 42 | **1.2 (1.1-1.3)** | | **1.1 (1.0-1.2)** | |  |
| Sex |  |  |  | |  | |  |  | |  | |  |  | |  | |  |
| Male | 744 | 22 | 1.0 | | Not retained | | 13 | 1.0 | | Not retained | | 38 | 1.0 | | Not retained | |  |
| Female | 43 | 1 | 0.8 (0.1 - 5.9) | |  | | 1 | 1.3 (0.2-10.5) | |  | | 4 | 1.9 (0.6-5.6) | |  | |  |
| Race / Ethnicity |  |  |  | |  | |  |  | |  | |  |  | |  | |  |
| White (non-Hispanic) | 470 | 3 | 1.0 | | Not retained | | 6 | 1.0 | | Not retained | | 10 | 1.0 | | Not retained | |  |
| Black (non-Hispanic) | 128 | 4 | **5.0 (1.1-22.7)** | |  | | 2 | 1.2 (0.2-6.2) | |  | | 7 | 2.7 (1.0-7.1) | |  | |  |
| Asian or Pacific Islander | 52 | 11 | **41.8 (11.2-155.7)** | |  | | 4 | **6.4 (1.8-23.6)** | |  | | 18 | **24.4 (10.4-56.9)** | |  | |  |
| Hispanic | 114 | 5 | **7.1 (1.7-30.3)** | |  | | 2 | 1.4 (0.3-6.9) | |  | | 7 | **3.0 (1.1-8.1)** | |  | |  |
| Native American or other | 23 | 0 | NA | |  | | 0 | NA | |  | | 0 | NA | |  | |  |
| TB prevalence in country of birth |  |  |  | |  | |  |  | |  | |  |  | |  | |  |
| <20 cases per 100,000 pop. | 713 | 6 | 1.0 | | 1.0 | | 9 | 1.0 | | 1.0 | | 16 | 1.0 | | 1.0 | |  |
| 20-100 cases per 100,000 pop. | 23 | 3 | **17.7 (4.1-75.8)** | | **14.4 (3.2-64.2)** | | 1 | 3.6 (0.4-29.3) | | 3.6 (0.4-29.3) | | 5 | **12.1 (4.0-36.6)** | | **12.4 (3.9-39.6)** | |  |
| >100 cases per 100,000 pop. | 51 | 14 | **44.6 (16.2-122.6)** | | **38.6 (13.7-109.0)** | | 4 | **6.7 (2.0-22.4)** | | **7.0 (2.1-23.5)** | | 21 | **30.5 (14.5-64.3)** | | **34.7 (15.2-79.2)** | |  |
| Highest TB prevalence in country of residence ^c^ |  |  |  | |  | |  |  | |  | |  |  | |  | |  |
| <20 cases per 100,000 pop. | 701 | 17 | 1.0 | | Not retained | | 12 | 1.0 | | Not retained | | 34 | 1.0 | | Not retained | |  |
| 20-100 cases per 100,000 pop. | 50 | 0 | NA | |  | | 0 | NA | |  | | 1 | 0.4 (0.1-3.0) | |  | |  |
| >100 cases per 100,000 pop. | 34 | 5 | **6.9 (2.4-20.1)** | |  | | 2 | 3.6 (0.8-16.7) | |  | | 6 | **4.2 (1.6-10.8)** | |  | |  |
| Residence or employment >1 month in high-risk congregate living facility ^d^ |  |  | |  | |  |  | |  | |  |  | |  | |  | |
| No | 639 | 19 | | 1.0 | | Not retained | 11 | | 1.0 | | Not retained | 32 | | 1.0 | | Not retained | |
| Yes | 147 | 4 | | 0.9 (0.3-2.7) | |  | 3 | | 1.12 (0.3 – 4.3) | |  | 10 | | 1.4 (0.7-2.9) | |  | |
| Reported TB exposure |  |  | |  | |  |  | |  | |  |  | |  | |  | |
| No | 764 | 22 | | 1.0 | | Not retained | 13 | | 1.0 | | Not retained | 40 | | 1.0 | | Not retained | |
| Yes | 23 | 1 | | 1.5 (0.2-11.9) | |  | 1 | | 2.6 (0.3- 21.0) | |  | 2 | | 1.7 (0.4 7.6) | |  | |
| History of BCG vaccination |  |  | |  | |  |  | |  | |  |  | |  | |  | |
| None | 708 | 11 | | 1.0 | | Not retained | 11 | | 1.0 | | Not retained | 26 | | 1.0 | | Not retained | |
| Unknown | 61 | 7 | | **8.2 (3.1-22.0)** | |  | 2 | | 2.1 (0.5-9.9) | |  | 9 | | **4.5 (2.0-10.2)** | |  | |
| Vaccinated | 18 | 5 | | **24.4 (7.4-80.2)** | |  | 1 | | 3.7 (0.5-30.5) | |  | 7 | | **16.7 (6.0-46.5)** | |  | |
| Reactivity to *M. avium* PPD |  |  | |  | |  |  | |  | |  |  | |  | |  | |
| No | 725 | 19 | | 1.0 | | NR | 12 | | 1.0 | | Not retained | 32 | | 1.0 | | 1.0 | |
| Yes | 62 | 4 | | 2.6 (0.8-7.8) | |  | 2 | | 2.0 (0.4-9.1) | |  | 10 | | **4.2 (1.9-8.9)** | | **6.8 (2.6-17.5)** | |

N = total number of recruits with completed test and determinate results; n = the number or recruits with positive test results; QFT-GIT = QuantiFERON®-TB Gold In-Tube test; TST = tuberculin skin test; OR (95% CI) = Odds Ratios (95% confidence intervals) with boldface font indicating statistically significant differences; aOR (95% CI) = adjusted Odds Ratios; BCG = bacille Calmette-Guérin; TB = tuberculosis. ^a^ TST induration >10 mm includes reactions >15 mm; ^b^ Increase in odds for each year of age; ^c^ Unknown for 2 subjects with other risk for *M. tuberculosis* infection; ^d^ Unknown for 1 subject with other risk for *M. tuberculosis* infection.
