# Supplementary material for: Assessment of the QuantiFERON-TB Gold In-Tube test for the detection of Mycobacterium tuberculosis infection in United States Navy recruits
Source: PLoS One. 2017 May 17;12(5):e0177752. doi: 10.1371/journal.pone.0177752 (PMC5435309; doi:10.1371/journal.pone.0177752)
Supplement: S3 Table — (DOCX) [file pone.0177752.s003.docx]

**S3 Table. Associations between selected subject characteristics and discordant QuantiFERON^®^-TB Gold In-Tube test and tuberculin skin test results using either a 15 mm or 10 mm cutoff.**

|  | TST > 15 mm but negative QFT-GIT | | | | TST > 10 mm but negative QFT-GIT | | | | TST <15 mm but positive QFT-GIT ^a^ | | |
| --- | --- | --- | --- | --- | --- | --- | --- | --- | --- | --- | --- |
| Characteristic | n ^Concord^ | n ^Discord^ | OR (95% CI) | aOR (95% CI) | n ^Concord^ | n ^Discord^ | OR (95% CI) | aOR (95% CI) | n ^Concord^ | n ^Discord^ | OR (95% CI) |
| Age ^b^ | 760 | 18 | **1.2 (1.1–1.3)** | **1.2 (1.0–1.3)** | 741 | 37 | **1.2 (1.1–1.3)** | 1.1 (1.0-1.2) | 760 | 9 | 1.1 (0.9-1.3) |
| Sex |  |  |  |  |  |  |  |  |  |  |  |
| Male | 719 | 17 | 1.0 | Not retained | 703 | 33 | 1.0 | Not retained | 719 | 8 |  |
| Female | 41 | 1 | 1.0 (0.1–7.9) |  | 38 | 4 | 2.2 (0.8-6.7) |  | 41 | 1 | 2.2 (0.3-17.9) |
| Race / Ethnicity |  |  |  |  |  |  |  |  |  |  |  |
| White (non-Hispanic) | 463 | 2 | 1.0 | Not retained | 456 | 9 | 1.0 | Not retained | 463 | 5 | 1.0 |
| Black (non-Hispanic) | 122 | 4 | **7.5 (1.4–41.9)** |  | 119 | 7 | **3.0 (1.1-8.2)** |  | 122 | 2 | 1.5 (0.3-7.9) |
| Asian or Pacific Islander | 45 | 7 | **36.0 (7.3–178.5)** |  | 38 | 14 | **18.7 (7.6-45.9)** |  | 45 | 0 | 0.0 |
| Hispanic | 107 | 5 | **10.8 (2.1–56.5)** |  | 105 | 7 | **3.4 (1.2-9.3)** |  | 107 | 2 | 1.7 (0.3-9.0) |
| Other | 23 | 0 | 0.0 |  | 23 | 0 | 0.0 |  | 23 | 0 | 0.0 |
| TB prevalence in birth country |  |  |  |  |  |  |  |  |  |  |  |
| <20 cases per 100,000 pop. | 700 | 5 | 1.0 | 1.0 | 690 | 15 | 1.0 | 1.0 | 700 | 8 | 1.0 |
| 20-100 cases per 100,000 | 19 | 3 | **22.1 (4.9–99.3)** | **18.0 (3.9-83.8)** | 17 | 5 | **13.5 (4.4-41.5)** | **13.5 (4.2-43.6)** | 19 | 1 | 4.6 (0.5-38.7) |
| >100 cases per 100,000 pop. | 41 | 10 | **34.1 (11.2–104.5)** | **27.8 (8.8-88.0)** | 34 | 17 | **23.0 (10.6-49.9)** | **24.2 (10.4-56.4)** | 41 | 0 | 0.0 |
| Highest TB prevalence in countries of residence ^c^ |  |  |  |  |  |  |  |  |  |  |  |
| <20 cases per 100,000 pop. | 680 | 13 | 1.0 | Not retained | 663 | 30 | 1.0 | Not retained | 680 | 8 | 1.0 |
| 20-100 cases per 100,000 | 50 | 0 | 0.00 |  | 49 | 1 | 0.5 (0.1-3.4) |  | 50 | 0 | 0.0 |
| >100 cases per 100,000 pop. | 29 | 4 | **7.2 (2.2–23.5)** |  | 28 | 5 | 3.9 (1.4-10.9) |  | 29 | 1 | 2.9 (0.4-24.2) |
| Residence or employment >1 month in high-risk congregate living facility ^d^ |  |  |  |  |  |  |  |  |  |  |  |
| No | 617 | 15 | 1.0 | Not retained | 604 | 28 | 1.0 | Not retained | 617 | 7 | 1.0 |
| Yes | 142 | 3 | 0.9 (0.2-3.0) |  | 136 | 9 | 1.4 (0.7-3.1) |  | 142 | 2 | 1.2 (0.3-6.0) |
| Reported TB exposure |  |  |  |  |  |  |  |  |  |  |  |
| No | 739 | 17 | 1.0 | Not retained | 721 | 35 | 1.0 | Not retained | 739 | 8 | 1.0 |
| Yes | 21 | 1 | 2.1 (0.3–16.3) |  | 20 | 2 | 2.1 (0.5-9.2) |  | 21 | 1 | 4.4 (0.5-36.8) |
| History of BCG vaccination |  |  |  |  |  |  |  |  |  |  |  |
| None | 690 | 9 | 1.0 | Not retained | 675 | 24 | 1.0 | Not retained | 690 | 9 | 1.0 |
| Unknown | 56 | 5 | **6.8 (2.2–21.1)** |  | 54 | 7 | **3.6 (1.5-8.8)** |  | 56 | 0 | 0.0 |
| Vaccinated | 14 | 4 | **21.9 (6.0–79.7)** |  | 12 | 6 | **14.1 (4.9-40.6)** |  | 14 | 0 | 0.0 |
| Reactivity to *M. avium* PPD |  |  |  |  |  |  |  |  |  |  |  |
| No | 702 | 15 | 1.0 | Not retained | 689 | 28 | 1.0 | 1.0 | 702 | 8 | 1.0 |
| Yes | 58 | 3 | 2.4 (0.7–8.6) |  | 52 | 9 | **4.3 (1.9-9.5)** | **6.2 (2.4-16.1)** | 58 | 1 | 1.5 (0.2-12.3) |

Table 6 Legend: QFT-GIT = QuantiFERON®-TB Gold In-Tube test; TST = tuberculin skin test; n ^Concord^ = the number of subjects with concordant TST (at indicated cutoff) and QFT-GIT results; OR (95% CI) = Odds Ratios (95% confidence intervals) with boldface font indicating statistically significant differences; BCG = bacille Calmette-Guérin; TB = tuberculosis. ^a^ TST induration was 0 mm for all subjects with TST <15 mm but positive QFT-GIT results so that all had TST-negative but QFT-GIT-positive discordance regardless of the TST cutoff used; ^b^ Increase in odds for each year of age; ^c^ Unknown for 2 subjects with other risk for *M. tuberculosis* infection; ^d^ Unknown for 1 subject with other risk for *M. tuberculosis* infection.
